# Value of lung diffusing capacity for nitric oxide in systemic sclerosis

**DOI:** 10.14814/phy2.14149

**Published:** 2019-07-01

**Authors:** Giovanni Barisione, Alessandro Garlaschi, Mariaelena Occhipinti, Michele Baroffio, Massimo Pistolesi, Vito Brusasco

**Affiliations:** ^1^ Unità Operativa Fisiopatologia Respiratoria Dipartimento di Medicina Interna Università di Genova Genova Italy; ^2^ Dipartimento della Diagnostica per Immagini e Radioterapia Ospedale Policlinico San Martino ‐ IRCCS Genova Italy; ^3^ Dipartimento di Medicina Sperimentale e Clinica Azienda Ospedaliero‐Universitaria Careggi Firenze Italy

**Keywords:** Interstitial lung disease, lung diffusing capacity for carbon monoxide, lung diffusing capacity for nitric oxide, systemic sclerosis

## Abstract

A decreased lung diffusing capacity for carbon monoxide (DL_CO_) in systemic sclerosis (SSc) is considered to reflect losses of alveolar membrane diffusive conductance for CO (DM_CO_), due to interstitial lung disease, and/or pulmonary capillary blood volume (V_C_), due to vasculopathy. However, standard DL_CO_ does not allow separate DM_CO_ from V_C_. Lung diffusing capacity for nitric oxide (DL_NO_) is considered to be more sensitive to decrement of alveolar membrane diffusive conductance than DL_CO_. Standard DL_CO_ and DL_NO_ were compared in 96 SSc subjects with or without lung restriction. Data showed that DL_NO_ was reduced in 22% of subjects with normal lung volumes and DL_CO_, whereas DL_CO_ was normal in 30% of those with decreased DL_NO_. In 30 subjects with available computed tomography of the chest, both DL_CO_ and DL_NO_ were negatively correlated with the extent of pulmonary fibrosis. However, DL_NO_ but not DL_CO_ was always reduced in subjects with ≥ 5% fibrosis, and also decreased in some subjects with < 5% fibrosis. DM_CO_ and V_C_ partitioning and Doppler ultrasound‐determined systolic pulmonary artery pressure could not explain individual differences in DL_CO_ and DL_NO_. DL_NO_ may be of clinical value in SSc because it is more sensitive to DM_CO_ loss than standard DL_CO_, even in nonrestricted subjects without fibrosis, whereas DL_CO_ partitioning into its subcomponents does not provide information on whether diffusion limitation is primarily due to vascular or interstitial lung disease in individual subjects. Moreover, decreased DL_CO_ in the absence of lung restriction does not allow to suspect pulmonary arterial hypertension without fibrosis.

## Introduction

Systemic sclerosis (SSc) is a generalized fibrotic disease classified among the autoimmune connective‐tissue disorders (van den Hoogen et al. 2013). Widespread microangiopathy (Asano and Sato 2015) and interstitial lung disease (ILD) (Baldwin Ede et al. 1949; Schoenfeld and Castelino 2015) are commonly found, whereas pulmonary arterial hypertension (PAH) affects only 5–12% of patients (Khanna et al. 2013). The impairment of lung function is generally inferred from measurements of forced vital capacity (FVC) and lung diffusing capacity for carbon monoxide (DL_CO_) (Schneider et al. 1982). However, FVC is not an accurate measure of lung restriction, that is, decreased total lung capacity (TLC) (Aaron et al. 1999; Pellegrino et al. 2005), and decrement of DL_CO_ in SSc may reflect loss of alveolar surface or thickening of blood‐gas barrier (Schoenfeld and Castelino 2015), but also changes in pulmonary microvasculature (Asano and Sato 2015).

Pulmonary CO uptake is mostly limited by its reaction rate with blood hemoglobin (Hb), thus DL_CO_ is considered to be more sensitive to changes of pulmonary capillary blood volume (V_C_) than alveolar membrane diffusive conductance for CO (DM_CO_) (Guénard et al. 1987; Borland and Higenbottam 1989). Accordingly, the reduced DL_CO_ in isolation or disproportionately to FVC seen in a minority of SSc subjects has been interpreted as a sign of PAH with limited or no ILD (Wilson et al. 1964; Steen et al. 1992), though the correlation between DL_CO_ and mean pulmonary artery pressure is weak (Mukerjee et al. 2004).

Nitric oxide (NO) has a much greater affinity and faster reaction with free Hb than CO (Gibson and Roughton 1957), which makes the lung diffusing capacity for nitric oxide (DL_NO_) presumably more sensitive to alveolar membrane diffusive conductance than DL_CO_ (Guénard et al. 1987; Borland and Higenbottam 1989). Thus, the analysis of combined DL_NO_ and DL_CO_ measurement has been proposed as a method to partition DM_CO_ and V_C_ subcomponents (Guénard et al. 1987) in various parenchymal (Phansalkar et al. 2004; Barisione et al. 2014, 2016) and vascular (Farha et al. 2013) disorders. By using this method in SSc, one study (Sivova et al. 2013) suggested that partitioning of DL_CO_ might be of interest to detect PAH either with or without ILD, whereas two other studies (Guarnieri et al. 2015; Degano et al. 2017) concluded that partitioning of DL_CO_ is of little use in distinguish the patients with only ILD from those with ILD complicated by PAH. Two studies (Overbeek et al. 2008; Pernot et al. 2012) using the classical multistep alveolar O_2_ partial pressure (PAO_2_) method (Roughton and Forster 1957) to calculate DM_CO_ and V_C_ also gave contrasting results. Although DL_NO_ has been demonstrated to be more sensitive than DL_CO_ in idiopathic pulmonary fibrosis and this was due to the prevailing DM_CO_ impairment (Barisione et al. 2016), this information in SSc is lacking. Therefore, the added value of DL_NO_ and the partitioning of diffusion subcomponents in the diagnostic workup of SSc is still unclear.

The aim of the present study was to investigate whether DL_NO_ and standard DL_CO_ can provide different information on the lung involvement in individuals with SSc that can be attributed to interstitial or vascular abnormalities by DM_CO_ and V_C_ partitioning.

## Materials and Methods

### Study subjects

We retrospectively collected data from 96 Caucasian consecutive subjects fulfilling the current diagnostic criteria for SSc with a total score ≥ 9 (van den Hoogen et al. 2013). They had pulmonary function and other diagnostic tests completed between March 2014 and October 2017, when they were in stable clinical conditions. Sixteen of them received long‐term intravenous iloprost, with various combinations of oral prednisone (*n* = 11), bosentan (*n* = 9), mofetil mycophenolate (*n* = 6), methotrexate (*n* = 4), and nifedipine (*n* = 3). Thirty‐nine healthy subjects, matched for anthropometric and life‐style data, were selected among health‐care professionals to serve as a control group (Table 1). The study was approved by the Regional Ethics Committee (Registry no.: P.R.170REG/2015) and written informed consent was obtained from each subject to use his/her personal data.

**Table 1:** Subjects’ anthropometric characteristics and lung function data

	Controls	SSc‐N	SSc‐D	SSc‐R	*P* value
Male/Female	5/34	2/49	1/18	5/21	0.24
Age (years)	55 ± 19	64 ± 13*, †	63 ± 10	54 ± 16†	0.008
BMI (kg·m^−2^)	25 ± 5	24 ± 4	24 ± 5	26 ± 5	0.27
Smoking habit (c/f/n)	4/6/29	3/6/42	5/5/9	1/6/19	0.08
[Hb] (g·dL^−1^)	13.5 ± 0.66	13.1 ± 0.93	12.7 ± 1.09*	12.9 ± 1.09	0.011
FVC (L)	3.53 ± 0.78	3.00 ± 0.54*	2.83 ± 0.68*	2.38 ± 0.63*, †, ‡	<0.001
(% predicted)	112 ± 14	104 ± 14*	96 ± 11*, †	66 ± 11*, †, ‡	<0.001
(*z*‐score)	0.72 ± 0.84	0.22 ± 0.89*	−0.31 ± 0.73*, †	−2.48 ± 0.93*, †, ‡	<0.001
FEV_1_ (L)	2.81 ± 0.68	2.31 ± 0.43*	2.17 ± 0.49*	1.97 ± 0.56*, †	<0.001
(% predicted)	110 ± 13	102 ± 12*	93 ± 12*, †	68 ± 9*, †, ‡	<0.001
(*z*‐score)	0.70 ± 0.83	0.10 ± 0.83*	−0.46 ± 0.78*, †	−2.23 ± 0.70*, †, ‡	<0.001
TLC (L)	5.25 ± 0.90	4.84 ± 0.59*	4.50 ± 0.99*	3.60 ± 0.83*, †, ‡	<0.001
(% predicted)	109 ± 10	103 ± 11*	94 ± 10*, †	67 ± 11*, †, ‡	<0.001
(*z*‐score)	0.66 ± 0.74	0.20 ± 0.86*	−0.40 ± 0.77*, †	−2.79 ± 0.94*, †, ‡	<0.001
DL_CO_ (mL·min^−1^·mmHg^−1^)	25.5 ± 6.12	18.2 ± 2.90*	12.4 ± 2.28*, †	14.5 ± 4.15*, †	<0.001
(% predicted)	116 ± 14	96 ± 12*	65 ± 10*, †	65 ± 12*, †	<0.001
(*z*‐score)	0.87 ± 0.72	−0.30 ± 0.70*	−2.73 ± 1.16*, †	−2.74 ± 1.17*, †	<0.001
K_CO_ (mL·min^−1^·mmHg^−1^·L^−1^)	4.60 ± 0.77	3.96 ± 0.56*	3.01 ± 0.53*, †	4.17 ± 0.65‡	<0.001
(% predicted)	104 ± 14	92 ± 12*	70 ± 12*, †	96 ± 14‡	<0.001
(*z*‐score)	0.24 ± 0.87	−0.52 ± 0.84*	−1.87 ± 1.35*, †	−0.31 ± 0.95‡	<0.001
DL_NO_ (mL·min^−1^·mmHg^−1^)	95.6 ± 26.0	71.7 ± 16.0*	52.5 ± 11.4*, †	55.9 ± 21.3*, †	<0.001
(% predicted)	88 ± 9	74 ± 11*	53 ± 9†*, †	46 ± 12*, †	<0.001
(*z*‐score)	−0.69 ± 0.53	−1.30 ± 0.66*	−2.35 ± 0.63*, †	−3.17 ± 0.88*, †, ‡	<0.001
K_NO_ (mL·min^−1^·mmHg^−1^·L^−1^)	18.8 ± 3.18	15.3 ± 2.38*	12.7 ± 2.64*, †	15.2 ± 3.40*, ‡	<0.001
(% predicted)	86 ± 9	74 ± 8*	61 ± 12*, †	68 ± 12*, †, ‡	<0.001
(*z*‐score)	−1.05 ± 0.71	−1.90 ± 0.56*	−2.91 ± 0.97*, †	−2.50 ± 0.98*	<0.001

Data are absolute numbers or mean ± SD.

SSc‐N, subjects with all standard lung function data > 5^th^ percentile (LLN_5_); SSc‐D, subjects with DL_CO_ < LLN_5_ but TLC > LLN_5_; SSc‐R, subjects with TLC < LLN_5_ with (*n* = 21) or without (*n* = 5) DL_CO_ < LLN_5_; c/f/n, current/former/never; FVC, forced vital capacity; FEV_1_, forced expiratory volume in one second; TLC, total lung capacity; DL_CO_, standard single‐breath (9‐11 s breath‐hold time) lung diffusing capacity for carbon monoxide; K_CO_, DL_CO_/alveolar volume; DL_NO,_ lung diffusing capacity for nitric oxide; K_NO_, DL_NO_/V_A_.

* Significantly different from control.

† Significantly different from SSc‐N.

‡ Significantly different from SSc‐D.

### Standard lung function measurements

Spirometry (Miller et al. 2005) and lung volumes (Wanger et al. 2005) were measured with the subject sitting in a whole‐body plethysmograph (V62J, SensorMedics‐Viasys, CareFusion; Höchberg, Germany). FVC, forced expiratory volume in one second (FEV_1_), FEV_1_/FVC ratio, and TLC were determined and compared with available predicted values (Quanjer et al. 1993, 2012). Standard DL_CO_ was measured (MasterScreen PFT System, Jaeger‐Viasys, CareFusion, Höchberg, Germany) by single‐breath technique with 9–11 breath‐hold time free of Valsalva or Müller maneuvers (Macintyre et al. 2005). CO back pressure was measured in expired gas prior to the inspiration of test mixture and compensated for analytically (Graham et al. 2002). Measured values were compared with reference values from Stanojevic et al. (2017) and adjusted for effective [Hb] in venous blood (Cotes et al. 1972).

### DL_NO_ measurement

At least 5‐10 min after standard DL_CO_, single‐breath DL_NO_‐DL_CO_ were simultaneously measured in sitting position (MasterScreen PFT System, Jaeger‐Viasys, CareFusion, Höchberg, Germany) from the exponential disappearance rate of NO and CO with respect to helium (He) in exhaled air (Guénard et al. 1987). A gas mixture of 0.28% CO, 9.0% He, and 20.9% O_2_ balanced with N_2_ was blended with 450 ppm NO in N_2_ and inhaled from a plastic bag containing a final concentration of NO of 50.1 ± 4.8 ppm obtained ≤ 2 min before its use. The linearity of the electrochemical cell was checked by factory and the apparatus was calibrated for gas fractions using automated procedures. The subject was in a relaxed sitting position, wearing a nose‐clip and breathing quietly through a mouthpiece with filter connected to a screen‐type pneumotachograph. After breathing 4–5 stable tidal volumes, he/she was requested to perform a full expiration to RV. Then a valve was opened allowing the subject to forcefully inhale the gas mixture achieving an inspired volume ≥ 90% of inspiratory vital capacity in < 2.5 sec. A breath‐hold time of 4–6 sec duration, at near atmospheric intrapulmonary pressure and free of Valsalva and Müller maneuvers, was then requested followed by a rapid and smooth expiration to RV (Zavorsky et al. 2017). The total breath‐hold time was calculated from the beginning of inspiration, minus 30% of inspiratory time, to the middle of expiratory gas sampling (Jones and Meade 1961). The first 750 mL of expired gas was discarded and the following 750 mL was sampled from the bag to be analyzed for NO, CO, and He fractional concentration. When the subject's vital capacity was < 2.0 L, sample and washout volumes were reduced to 500 mL (Macintyre et al. 2005). Between successive tests, an interval of ≥ 4–5 min was allowed to ensure complete elimination of prior test gases from the lungs. DL_NO_‐DL_CO_ values were accepted if two successive measurements were within 17.0 and 3.2 mL·min^−1^·mmHg^−1^, respectively, and the mean of two properly performed maneuvers was retained for analysis (Zavorsky and Murias 2006).

### Derivation of diffusion subcomponents

DM_CO_ and V_C_ were derived by assuming a fixed value of blood conductance for NO (*θ*_NO_) of 4.5 mL (NO)·min^−1^·mmHg^−1^·mL (blood)^−1^ (Carlsen and Comroe 1958) which is largely independent of pulmonary blood [Hb] (van der Lee et al. 2005) and mean (expired) PAO_2_ $\overset{¯}{P}{\text{AO}}_{2}$ (Borland and Cox 1991). By contrast, the value of blood conductance for CO (*θ*_CO_) is variable and strictly dependent on [Hb] (Cotes et al. 1972) and $\overset{¯}{P}{\text{AO}}_{2}$, due to the competitive binding of CO and O_2_ for Hb‐accessible sites (Crapo et al. 1988). This makes the inspired O_2_ fractional concentration (F_I_O_2_) critical to DL_CO_ measurement (Crapo et al. 1988) and, in turn, the DM_CO_ and V_C_ partitioning (Roughton and Forster 1957; Forster 1987). Thus, *θ*_CO_ values were calculated according to Guénard et al.(2016) in vivo equation:

(1) $$\frac{1}{\theta_{\mathrm{co}}}=1.16+0.0062\cdot \overset{¯}{P}{\text{bO}}_{2}\cdot \left(\frac{\left[Hb_{\mathrm{stand}}\right]}{\left[Hb_{\mathrm{meas}}\right]}\right)$$

where capillary O_2_ partial pressure ($\overset{¯}{P}{\mathrm{bO}}_{2}$) is equal to [$\mathrm{PA}O_{2}-\left(\dot{V}O_{2}/DL_{O_{2}}\right)$] (Bohr 1909), with $\dot{V}{\text{O}}_{2}/{\text{DL}}_{{\text{o}}_{2}}$ being the ratio of O_2_ uptake to lung diffusing capacity for O_2_ which was assumed as ≈DL_CO_·1.61 (Hsia et al. 2008). Moreover, [Hb] standard values $\left(\left[Hb_{\mathrm{stand}}\right]\right)$ were set at 14.6 and 13.4 g∙dL^‐1^ for males and females, respectively (Cotes et al. 1972). For the simultaneous DL_NO_‐DL_CO_ measurement, due to an automated flushing procedure with 100% O_2_ preceding each test and specific to this apparatus (Munkholm et al. 2018), the actual mean F_I_O_2_ (${\text{F}}_{I}{\text{O}}_{2}$) in the inspiratory bag was 0.228 ± 0.01, thus resulting in a $\overset{¯}{P}{\text{bO}}_{2}$ of 125.4 ± 8.5 mmHg instead of the expected 99.1 ± 3.3 mmHg. To account for this potential bias, the corresponding values of *θ*_CO_ were calculated from Equation (1) to be 0.50 ± 0.04 and 0.55 ± 0.04 mL (CO)∙min^−1^∙mmHg^−1^∙mL (blood)^−1^, with $\overset{¯}{P}{\text{bO}}_{2}$ of 125.4 ± 8.5 and 99.1 ± 3.3 mmHg, respectively. These values were used to derive the diffusion subcomponents in mild hyperoxia and normoxia from simultaneous DL_NO_‐DL_CO_ and separate standard DL_CO_ and DL_NO_ measurements, respectively, as follows (Zavorsky et al. 2017):

(2) $${\text{DM}}_{\mathrm{NO}}=\left(\frac{\theta_{\mathrm{NO}}-2\cdot \theta_{\mathrm{co}}}{\frac{\theta_{\mathrm{NO}}}{{\text{DL}}_{\mathrm{NO}}}-\frac{\theta_{\mathrm{co}}}{{\text{DL}}_{\mathrm{CO}}}}\right)\text{and}\;V_{C}\left(\frac{1}{\frac{\theta_{\mathrm{CO}}}{{\text{DL}}_{\mathrm{CO}}}-\frac{\theta_{\mathrm{co}}}{{\text{DM}}_{\mathrm{CO}}}}\right)$$

where DM_CO_ = DM_NO_/1.97 according to NO/CO physical diffusivity ratio (Wilhelm et al. 1977). Predicted values for DL_NO_ and diffusion subcomponents were from Zavorsky et al.(2017).

### Chest CT scanning

In 30 subjects, a thin‐section CT scan obtained within 3 months before or after pulmonary function measurements was available. Scans of the entire chest had been routinely obtained in a supine position, during breath‐hold at full inspiration by a multidetector row‐spiral scanner (SOMATOM Emotion 6, Siemens AG Medical, Forchheim, Germany). Images were acquired by 110 kVp tube voltage at 1.25 mm slice thickness and reconstructed at 1‐mm increments by using smooth (B41s) and sharp (B70s) convolution kernels. All CT scans were independently analyzed quantitatively as well as semi‐quantitatively by two experienced readers (G.B. and A.G.), with the latter being blind to clinical diagnosis and lung function data. For both analyses, the following six axial levels were considered: (1) aortic arch, (2) tracheal carina, (3) right pulmonary venous confluence, (4) midpoint between level three and level five, (5) 1 cm above the dome of the right hemi‐diaphragm, and (6) 2 cm below the dome of the right hemi‐diaphragm (Colombi et al. 2015). Mean lung density (g·mL^−1^) was calculated by adding CT numbers (HU) and parenchymal volume (Gattinoni et al. 2005) measured in 2D at the six axial levels. In 13 subjects, mean lung density could also be obtained by automatic quantitative 3D analysis of the entire lungs (VIDA's Lung Volume Analysis, Coralville, IA, US). At each axial level, ground glass opacities and fibrotic changes, that is, subpleural honeycombing, reticular opacities, and traction bronchiectasis, were visually bounded and their respective volumes computed by a three‐dimensional active contour segmentation software (ITK‐Snap 3.6.0, Philadelphia, PA, USA) (Yushkevich et al. 2006). In each patient, the total percentages of lung volume with ground glass attenuation and/or fibrotic changes were calculated in relation to the total parenchymal volume of the six axial levels (Barisione et al. 2016).

### Doppler echocardiography

In all subjects, a transthoracic Doppler echocardiography (Vivid E90, GE Healthcare, Little Chalfont, UK) was routinely obtained within 3 months before or after pulmonary function testing. In subjects with measurable tricuspid regurgitation velocity (TRV), systolic pulmonary artery pressure (sPap) was calculated as sPap = (4·TRV^2^ + RAP), where RAP is right atrial pressure, estimated from the diameter and breath‐induced variability in the inferior vena cava (Hatle et al. 1981). Values of sPap ≥ 36 mmHg were considered as suggestive of likely PAH (Greiner et al. 2014).

### Statistical analysis

Lung function data were expressed as *z*‐score, which indicates how many standard deviations a given measure differs from predicted, with a value −1.645, corresponding to the 5^th^ percentile of the reference population, assumed as the lower limit of normal (LLN_5_). Categorical variables were compared by *z*‐test with Yates correction and continuous variables by ANOVA with Holm‐Sidak post hoc test for multiple comparisons. Associations between variables were determined by Spearman's rank correlation. Data are presented as mean ± SD or median with 25–75% interquartile range (IQR_25–75%_). In all analyses, the acceptable type I error was set at *P *<* *0.05.

## Results

Based on the standard lung function data, SSc subjects as the whole (*n* = 96) were divided into three groups, one with both TLC and DL_CO_ above the LLN_5_ (SSc‐N) (*n* = 51), one with DL_CO_ below the LLN_5_ but TLC above the LLN_5_ (SSc‐D) (*n* = 19), and one with TLC below the LLN_5_ with or without DL_CO_ below the LLN_5_ (SSc‐R) (*n* = 26). Two SSc‐D subjects showed mild‐to‐moderate airflow obstruction, but no CT evidence of emphysema. Eighteen out of the 39 subjects with measurable TRV had CT scan analysis and nine of them showed sPap values > 36 mmHg. Although, by definition all subjects of SSc‐N group had standard lung function parameters above the LLN_5_, the group mean values were significantly (*P *<* *0.001) lower than in the control group.

**Figure 1:**
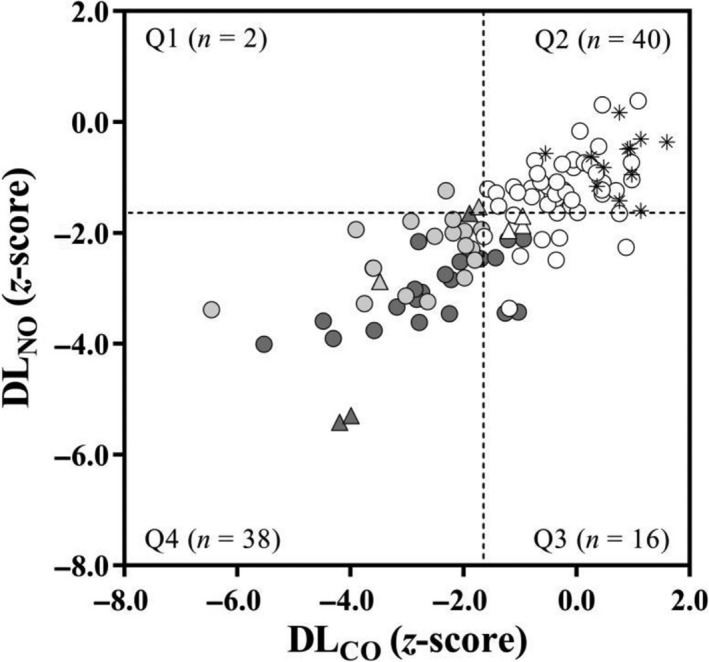
Relationships between the *z*‐scores of lung diffusing capacity for nitric oxide (DL_NO_) (*y*‐axis) and standard (9–11 sec breath‐hold time) lung diffusing capacity for carbon monoxide (DL_CO_) (*x*‐axis) in healthy controls (asterisks) and SSc subjects. Symbols indicate subjects with spirometry and total lung capacity (TLC) > LLN_5_ (white), DL_CO_ < LLN_5_ (light grey), and TLC < LLN_5_ (dark grey). Triangles indicate subjects with systolic pulmonary artery pressure (sPap) > 36 mmHg. The dashed lines indicate the LLN_5_ (*z*‐score < −1.645). The absolute values indicate the number of the total 96 test results for DL_NO_ that fall into each quadrant (Q1–Q4).

**Figure 2:**
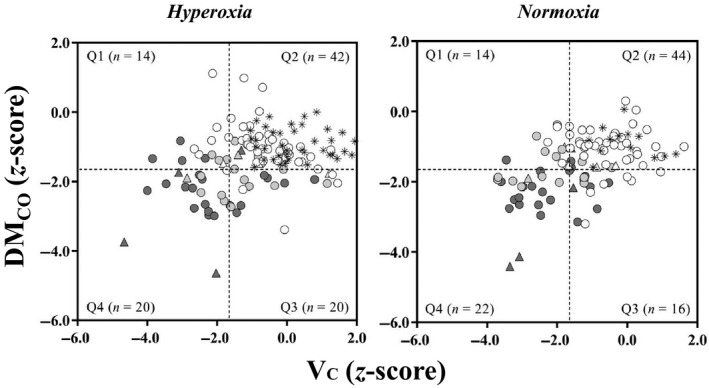
Relationships between the *z*‐scores of alveolar membrane diffusive conductance for CO (DM_CO_) (*y*‐axis) and pulmonary capillary blood volume (V_C_) (*x*‐axis) in mild hyperoxia (left panel) and normoxia (right panel) in healthy controls (asterisks) and SSc subjects. Symbols indicate subjects with spirometry and total lung capacity (TLC) > LLN_5_ (white), DL_CO_ < LLN_5_ (light grey), and TLC < LLN_5_ (dark grey). Triangles indicate subjects with systolic pulmonary artery pressure (sPap) > 36 mmHg. The dashed lines indicate the LLN_5_ (*z*‐score < −1.645). The absolute values indicate the number of the total 96 test results for DM_CO_ that fall into each quadrant (Q1–Q4).

Considering individual data, DL_NO_ was below the LLN_5_ in all restricted subjects, all but two SSc‐D, eight out of nine with sPap > 36 mmHg, and also 11 out of 51 SSc‐N subjects (Fig. 1). DL_CO_ was below the LLN_5_ in 21 out of 26 SSc‐R subjects and six out of nine with sPap > 36 mmHg. DM_CO_ was below the LLN_5_ in the majority but not all SSc‐R subjects and V_C_ was below the LLN_5_ in the majority but not all SSc‐D subjects (Fig. 2). Overall, using LLN_5_ as cutoff value, neither DL_NO_ nor DL_CO_ provided falsely positive results (100% specificity) but their sensitivities to the presence of SSc were 56% and 42%, respectively.

**Figure 3:**
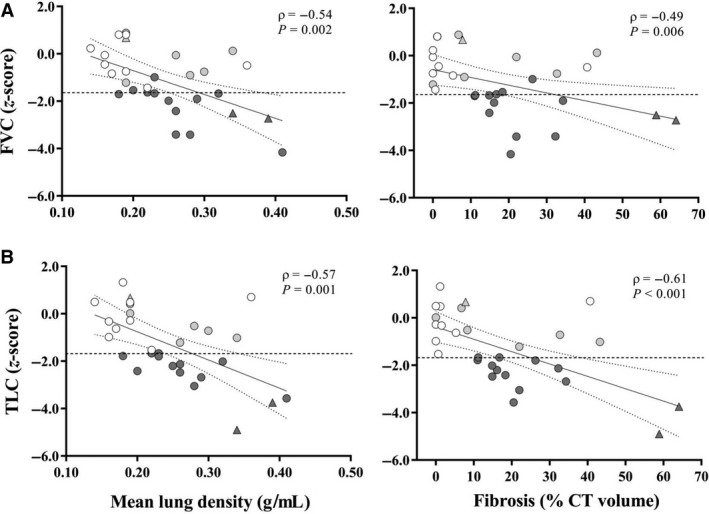
Correlations between mean lung density (g·mL^−1^) or fibrosis extent (% CT volume) and *z*‐scores of (A) forced vital capacity (FVC) and (B) TLC. Symbols indicate SSc subjects with spirometry and TLC > LLN_5_ (white), DL_CO_ < LLN_5_ (light grey), and TLC < LLN_5_ (dark grey) with (*n* = 11) or without (*n* = 3) DL_CO_ < LLN_5_. Triangles indicate subjects with sPap > 36 mmHg. CI_95%_ of the best‐fit regression line is marked by dotted lines whereas horizontal dashed line indicates the 5^th ^ percentile of reference values (−1.645 *z*‐score).

**Figure 4:**
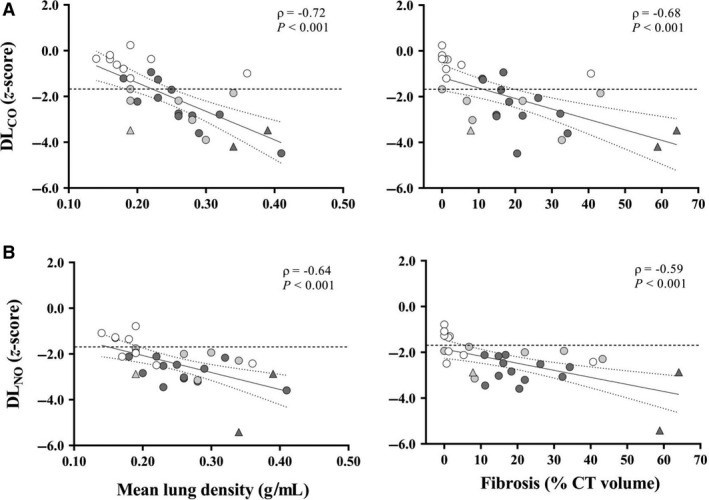
Correlations between mean lung density (g·mL^−1^) or fibrosis extent (% CT volume) and *z*‐scores of (A) standard DL_CO_ and (B) DL_NO_. Symbols indicate SSc subjects with spirometry and TLC > LLN_5_ (white), DL_CO_ < LLN_5_ (light grey), and TLC < LLN_5_ (dark grey) with (*n* = 11) or without (*n* = 3) DL_CO_ < LLN_5_. Triangles indicate subjects with sPap > 36 mmHg. CI_95%_ of the best‐fit regression line is marked by dotted lines whereas horizontal dashed line indicates the 5^th^ percentile of reference values (−1.645 *z*‐score).

In 30 subjects with analyzable CT scans, the median extent of fibrosis was 15% (IQR_25–75%_, 2 to 26%), while ground glass attenuation was 1% (IQR_25–75%_, 0 to 9%). As the between‐observer agreement of CT scans was very good for both fibrosis (weighted *K*, 0.92; 95% confidence interval, 0.84 to 0.99) and ground glass (weighted *K*, 0.82; 95% confidence interval, 0.70 to 0.94), the mean of two readings was retained for analysis. Both FVC and TLC *z*‐scores correlated significantly with mean lung density and fibrosis extent (Fig. 3A and B) but were above the LLN_5_ not only in subjects with < 5% fibrosis, but also in a consistent number (11 and 8, respectively) of those with ≥ 5% fibrosis. DL_CO_ and DL_NO_ also correlated significantly with mean lung density and fibrosis extent (Fig. 4A and B). However, DL_NO_ but not DL_CO_ was below the LLN_5_ in all subjects with ≥ 5% fibrosis. Moreover, DL_NO_ was below the LLN_5_ in four out of nine subjects with ≤ 5% fibrosis. There were no significant correlations between ground glass extent and any measure of lung function.

## Discussion

The main findings of the present study can be summarized as follows: (1) DL_NO_ was reduced in 56% of subjects with SSc and in 22% of those with normal lung volumes and standard DL_CO_, (2) standard DL_CO_ was normal in 30% of subjects with reduced DL_NO_, (3) DM_CO_ and V_C_ partitioning did not provide consistent explanations for individual differences in DL_NO_ and DL_CO_, and (4) both DL_NO_ and DL_CO_ were significantly correlated with CT measurements of ILD but only the former was consistently reduced in all subjects with fibrosis extent ≥ 5%.

### Comments on methodology

**Figure 5:**
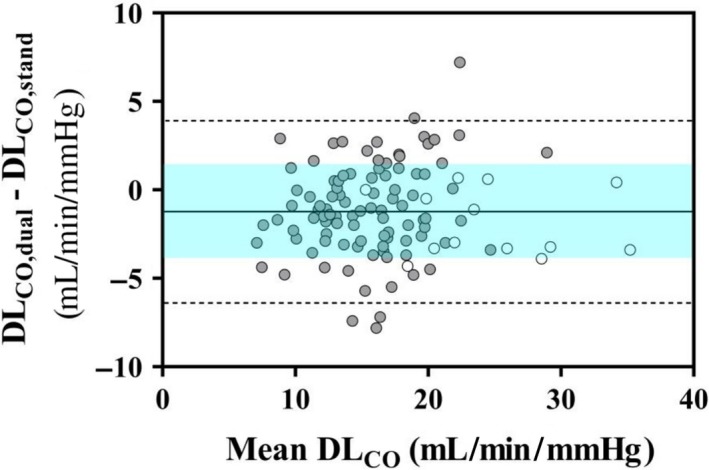
Bland‐Altman plot of the difference between absolute values of lung diffusing capacity for CO measured in mild hyperoxia (DL_CO,dual_) and normoxia (DL_CO,stand_) (*y*‐axis) vs. mean DL_CO_ value (*x*‐axis) in healthy controls (white circles) and SSc subjects (grey circles). The standard deviation (SD) of mean difference is bounded by the shaded area included between the horizontal dashed lines indicating 95% confidence interval (CI_95%_). It is noteworthy the scattered fluctuations of data around the mean value and the limits of agreement exceeding CI_95%_ in four cases.

**Figure 6:**
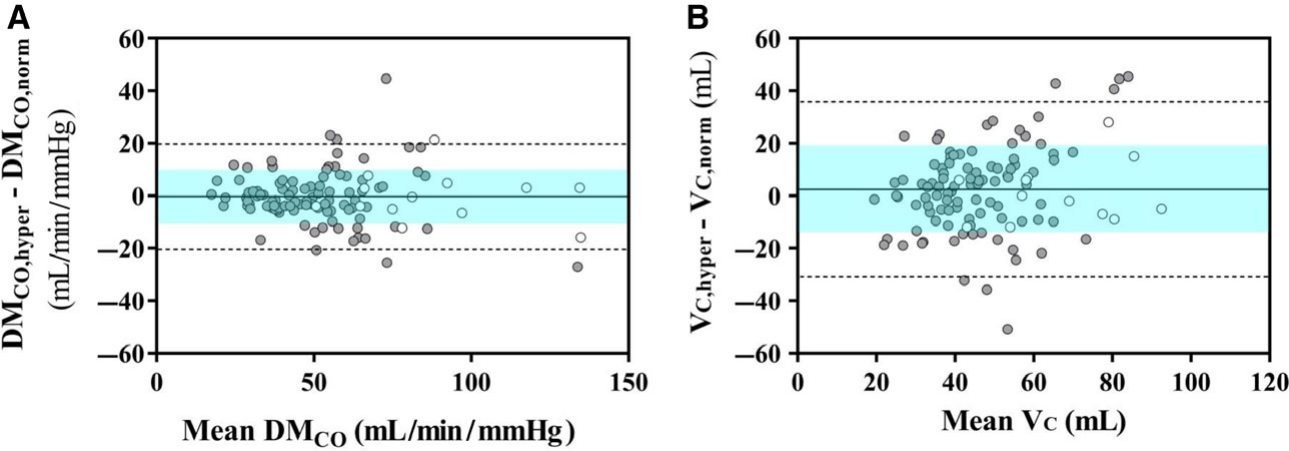
Bland‐Altman plots of the difference between absolute values of alveolar membrane diffusive conductance for CO (DM_CO_) (left panel) and pulmonary capillary blood volume (V_C_) (right panel) both derived in mild hyperoxia and normoxia versus their respective mean value (*x*‐axis) in healthy controls (white circles) and SSc subjects (grey circles). The SD of mean differences is bounded by the shaded areas included between the horizontal dashed lines indicating CI_95%_. It is noteworthy the scattered fluctuations of data around the mean value of the relevant parameter and the limits of agreement exceeding CI_95%_ in six and seven cases for DM_CO_ and V_C_, respectively.

To account for the possible bias due to an increased F_I_O_2_ (Munkholm et al. 2018) by an automatic flushing procedure with 100% O_2_ before each test in the commercially available system used in the present study, we determined DM_CO_ and V_C_ using DL_CO_ measured either in combination with DL_NO_ or separately by standard technique (Figs. 5 and 6). These required different breath‐hold times, that is, 4–6 and 9–11 sec, respectively, but this seems to have a negligible effect on final DL_CO_ values (Graham et al. 1985).

In contrast to previous studies (Overbeek et al. 2008; Pernot et al. 2012; Sivova et al. 2013; Guarnieri et al. 2015), lung function data were expressed as *z*‐scores, instead of absolute or percentage values, to avoid age‐, sex‐, and height‐biases. Although the 2.5^th ^ percentile (*z*‐score of −1.96) has been suggested as a lower limit of normal for DL_NO_ measurement (Zavorsky et al. 2017), we used the 5^th^ percentile (*z*‐score −1.645) in order to make the results comparable with standard DL_CO_ and avoid false negatives in subjects with established clinical diagnosis (Quanjer et al. 1993). On the other hand, no subject of the control group had DL_NO_, DM_CO_, or V_C_ *z*‐scores below −1.645, thus making the possibility of false positives unlikely.

### Study limitations

*First*, the whole group of our control subjects showed a DL_NO_ mean *z*‐score of ‐0.69 ± 0.53. However, we used the reference equation (Zavorsky et al. 2017) that provided the lowest SD (0.58) of *z*‐scores of our local dataset of healthy subjects. Moreover, all individual *z*‐scores were definitely above the quoted LLN_5_ (−1.645) for DL_NO_. *Second*, only 30 CT scans taken within 3 months from lung function studies were considered to reduce temporal variability and were analyzed at six axial levels. The CT images of 13 subjects were also suited for advanced quantitative analysis using all CT slices, and mean lung density was strongly correlated with that of the six axial levels (*r *=* *0.99; *P *<* *0.001). Thus, it seems reasonable to assume that the latter was representative of the whole lungs. *Third*, sPap was derived from TRV, which may not be detectable by transthoracic Doppler echocardiograpy in about half of subjects with invasively determined PAH (O'Leary et al. 2018). Although an estimated sPap > 50 mmHg has been arbitrarily recommended by previous guidelines to make PAH likely (Galiè et al. 2009), in a large recent study values ≥ 36 mmHg have been shown to provide a > 90% positive predictive value (Greiner et al. 2014). Nevertheless, PAH cannot be ruled out in subjects with undetected TRV. *Fourth*, while lung function tests were obtained in a sitting position, CT scans were acquired in supine posture, which might have altered V_C_ (Cotton et al. 1990) and possibly mean lung density. It seems, however, unlikely that changes in body position might have altered the extent of fibrotic changes. *Finally*, 40 ppm of NO in the inspired gas could decrease hypoxic pulmonary vasoconstriction (Asadi et al. 2015), but this was found for PAO_2_ values < 60 mmHg (Glenny and Robertson 2011), thus well below the values of 125.4 ± 8.5 and 99.1 ± 3.3 mmHg calculated for the hyperoxic and normoxic conditions of this study, respectively.

### Comments on results

The value of DL_CO_ partitioning into DM_CO_ and V_C_ subcomponents was investigated in five previous studies, two using the multistep PAO_2_ method (Overbeek et al. 2008; Pernot et al. 2012) and three using the DL_NO_‐DL_CO_ method (Sivova et al. 2013; Guarnieri et al. 2015; Degano et al. 2017). Altogether, these studies showed that DM_CO_ could be reduced in subjects with ILD, irrespective of the presence of PAH (Overbeek et al. 2008; Pernot et al. 2012; Sivova et al. 2013; Guarnieri et al. 2015; Degano et al. 2017), but also in subjects with isolated PAH without ILD (Guarnieri et al. 2015). By contrast, V_C_ was reduced in subjects with PAH without ILD (Guarnieri et al. 2015; Degano et al. 2017) but highly variable in subjects with ILD, whether associated with PAH (Overbeek et al. 2008; Pernot et al. 2012; Sivova et al. 2013; Guarnieri et al. 2015; Degano et al. 2017), or not (Overbeek et al. 2008; Pernot et al. 2012; Guarnieri et al. 2015; Degano et al. 2017). Although it has been suggested that DL_NO_ may be more sensitive than standard DL_CO_ in subjects without ILD or PAH (Guarnieri et al. 2015), none of the above studies reported direct comparisons between these two measures in individual subjects.

Consistent with previous investigations (Abramson et al. 1991; Steen et al. 1992; Jacobsen et al. 1997; Guarnieri et al. 2015; Degano et al. 2017), a substantial number of SSc subjects of the present study had standard lung function measurements, including DL_CO_, within their respective normal ranges, that is, the 90% confidence interval. New findings of this study are that DL_NO_ was reduced not only in all subjects with restriction, but also in 95% of those with reduced DL_CO_ but no restriction, and about 22% of those with otherwise normal lung function. Consistent with these findings is the reduction in DL_NO_ also observed in subjects with minimal or no fibrosis on CT scan. By converse, DL_CO_ was normal in 30% of subjects with reduced DL_NO_, including individuals with lung restriction or likely PAH. In keeping with this finding is the normal DL_CO_ also observed in some subjects with 10–40% fibrosis on CT scan. As 70–80% of the total resistance to CO uptake is deemed to be in the blood red cells (Guénard et al. 1987; Borland and Higenbottam 1989), a reduction in DL_CO_ without lung restriction has been considered as a sign of PAH (Wilson et al. 1964; Steen et al. 1992). Moreover, as the red cell resistance to NO uptake is only 40% of total (Borland et al. 2014), DL_CO_ should be expected to be reduced in subjects without restriction or ILD more than DL_NO_ and this difference be associated with V_C_ rather than DM_CO_ decrement. Neither of these predictions is supported by the present findings.

A possible reason for DL_NO_ being more sensitive than DL_CO_ even in the absence of restriction or fibrosis may be that DM_CO_ is not simply related to membrane thickness and alveolar surface but to total gas exchange area, including vessel and red cell surfaces, whereas V_C_ is related to pulmonary blood volume only (Kang and Sapoval 2016). Disproportionate reductions in gas exchange area may be the consequence of geometric heterogeneity of vascular bed, due to capillary remodeling or obliteration with blood volume being redistributed to unaffected lung regions, thus reducing DM_CO_ but not total V_C_ (Pande et al. 1975; Oppenheimer et al. 2006). DM_CO_ impairment might be also the consequence of uneven blood red cell distribution within the alveolar capillaries with enhanced erythrocyte clustering and deformation (Hsia et al. 1997) due to increased plasma viscosity (Spengler et al. 2004), thus reducing their surface but not volume (Oppenheimer et al. 2006). These mechanisms are in keeping with previous data from Farha et al. (2013) showing that decreased gas transfer in idiopathic PAH may be due to loss of either DM_CO_ or V_C_ and may explain the variability in results of the present study in subjects with sPap > 36 mmHg. Another reason for decreased DM_CO_ without V_C_ changes could be a thickening of alveolar‐to‐capillary membrane by the presence of interstitial edema. However, there was a strict correlation between mean lung density and % fibrosis extent (*r *=* *0.80; *P *<* *0.001, data not shown) and there was no significant ground glass attenuation in most of subjects, which makes this mechanism unlikely. On the other hand, owing to the strict dependence of DL_CO_ on specific blood *θ*_CO_ (Roughton and Forster 1957), a preserved V_C_ may attenuate the effects of alveolar‐to‐capillary membrane thickening by expanding the surface area available for gas exchange, which may explain the preserved DL_CO_ in subjects with lung restriction or fibrosis.

## Conclusions

Collectively, the results of the present study suggest that the measurement of DL_NO_ may be of clinical value in the diagnostic workup of SSc, because it is more sensitive than DL_CO_, either in the presence or absence of lung restriction or fibrosis. However, DM_CO_ and V_C_ partitioning does not seem to be useful to tell whether different results of DL_NO_ and DL_CO_ are primarily due to vascular or interstitial lung disease in individual subjects. Finally, decreased DL_CO_ in the absence of lung restriction does not allow to suspect PAH without ILD.

## Conflict of Interest

G.B., A.G., M.B. and M.P. have no financial/nonfinancial interests to disclose; M.O. received personal fees from Imbio LLC for consultancies and a grant for Ph.D. course from Menarini Foundation; V.B. received personal fees and nonfinancial support for consultancy, given lecture, and travel reimbursement from ndd Medizintechnik.
